# Transition from oncology to primary care: experience of long-term cancer survivors in a Brazilian high-complexity center

**DOI:** 10.1007/s00520-026-11034-w

**Published:** 2026-07-25

**Authors:** Lidiane Araujo Cezário, Luiza Vianna Conteville, Juliane Alves da Silva, Gabriela Villaça Chaves

**Affiliations:** 1 Departamento de Nutrição e Dietética, Hospital Do Câncer III (HC-III), Instituto Nacional de Câncer (INCA), Rua Visconde de Santa Isabel, Vila Isabel, Rio de Janeiro, 274 Brazil; 2 Programa de Pós-Graduação em Oncologia (PPGO-INCA), Rua André Cavalcanti, 37, Rio de Janeiro, Centro Brazil; 3 Divisão de Vigilância e Análise de Situação, Instituto Nacional de Câncer (INCA), Rua Marquês de Pompal, 125, Rio de Janeiro, Centro Brazil

**Keywords:** Cancer survivors, Endometrial neoplasms, Breast neoplasms, Prostatic neoplasms, Colorectal neoplasms, Primary health care

## Abstract

**Purpose:**

The aim of the present study was to describe access to primary health care, adherence to preventive and surveillance recommendations, and the prevalence of late effects among long-term cancer survivors referred back to primary health care (PHC) after five years of treatment and follow-up at an Oncology High Complexity Care Center (CACON).

**Methods:**

A descriptive cross-sectional study was conducted at the National Cancer Institute (INCA), in Brazil. Eligible participants were contacted by telephone and answered a structured questionnaire, with questions about sociodemographic data, lifestyle, and nutritional status, as well as on health promotion, prevention, and surveillance during the long-term cancer survivorship phase.

**Results:**

Ninety-one cancer survivors participated in the study, with an average age of 69 ± 9.24 years. The most commonly reported late sequelae included cognitive difficulties, hormonal symptoms, sleep-related disorders and chronic pain. Less than half of the study participants reported being referred to a PHC unit after cancer treatment. Receipt of a discharge summary to be presented to primary care providers was reported by only 11 participants (12.1%).

**Conclusions:**

The specific needs of long-term cancer survivors require continuous follow-up by the health system, aiming to ensure comprehensive health care and quality of life.

**Supplementary Information:**

The online version contains supplementary material available at 10.1007/s00520-026-11034-w.

## Introduction

Cancer is considered a chronic disease with impacts that may extend for years after diagnosis and treatment, whether they are physical, psychological, or social in nature [1]. The integrative care of cancer survivors should be a priority area of study and research in oncology since, even after achieving a cure, survivors frequently experience long-term treatment-related effects that affect their quality of life.

Although cancer survivors’ experiences vary according to cancer type and time since treatment, it is estimated that about 30.0% of individuals present at least one unresolved emotional, social, or spiritual concern [2]. Chronic symptoms or side effects are also associated with poor quality of life in this population [3]. Prostate cancer survivors, especially those with treatment-related sequelae, more frequently report problems related to sexuality and fear of cancer recurrence [4]. Colorectal cancer survivors report fatigue, financial issues, and psychological concerns [5]. Similar needs have also been described in endometrial [6] and breast cancer [7].

A study conducted in Brazil, from the perspective of patients and their caregivers, identified several consequences related to cancer treatment, reinforcing the relevance of specialized care that responds to the concrete challenges experienced by this population. Aspects such as improved access to health facilities, financial support during treatment, and attention to mental health were pointed out [8]. In this context, the long-term issues faced by cancer survivors demand adherence to recommendations related to surveillance, psychosocial support, and interventions aimed at health promotion and disease prevention [9].

The aim of the present study is to describe access to primary health care, adherence to preventive and surveillance recommendations, and the prevalence of late effects among long-term cancer survivors referred back to primary health care (PHC) after five years of treatment and follow-up at an Oncology High Complexity Care Center (CACON).

## Methods

### Study design and eligibility

This descriptive cross-sectional study was conducted at the National Cancer Institute (INCA) in Rio de Janeiro. Eligible participants were survivors of endometrial, prostate, colorectal, and breast cancers who had been discharged from the institution by May 2022, had completed their last cancer treatment between 2015 and 2018, and had no evidence of recurrence or disease progression for at least five years. Among eligible participants, reasons for non-participation included inability to establish telephone contact, refusal to participate, being deceased, and health conditions that prevented participation.

### Data collection

Eligible patients were contacted by telephone. Data collection began after formal consent was obtained through the Informed Consent Form via audio-recorded authorization.

A structured questionnaire specifically developed for this study was administered, including patient-reported measures. The questionnaire was designed based on a literature review on the topic, including the identification of needs reported by long-term cancer survivors in previous studies. Questions addressing health status, health behaviors, and healthcare utilization were adapted from previously validated instruments, including national health surveys, while additional items were developed to assess cancer survivorship-specific issues not covered by existing questionnaires. The selection of domains considered the Clinical Practice Guidelines in Oncology for Cancer Survivors from the National Comprehensive Cancer Network (NCCN) [10]. A pilot test of the questionnaire was conducted to evaluate the time required for its administration, question clarity, the presence of ambiguous or poorly worded items, and participants' feedback. The instrument was subsequently revised as needed.

The questionnaire included questions on sociodemographic data, lifestyle, and nutritional status, as well as on health promotion, prevention, and surveillance during the long-term cancer survivorship phase. The domains, along with their respective assessed dimensions, are described in Supplementary Table 1.

Information related to cancer diagnosis and treatment was obtained through direct data collection from both physical and electronic medical records.

### Statistical analysis

Statistical analyses were performed using SPSS version 25. For the analysis and presentation of results, measures of central tendency and dispersion were calculated for numerical variables, while proportion measures were calculated for categorical variables.

Differences between proportions were tested using Pearson’s chi-square test or Fisher’s exact test. The independent-samples Student’s *t*-test was used to compare differences between the means of numerical variables. For all analyses, a *p*-value < 0.05 was considered statistically significant.

## Results

**Fig. 1:**
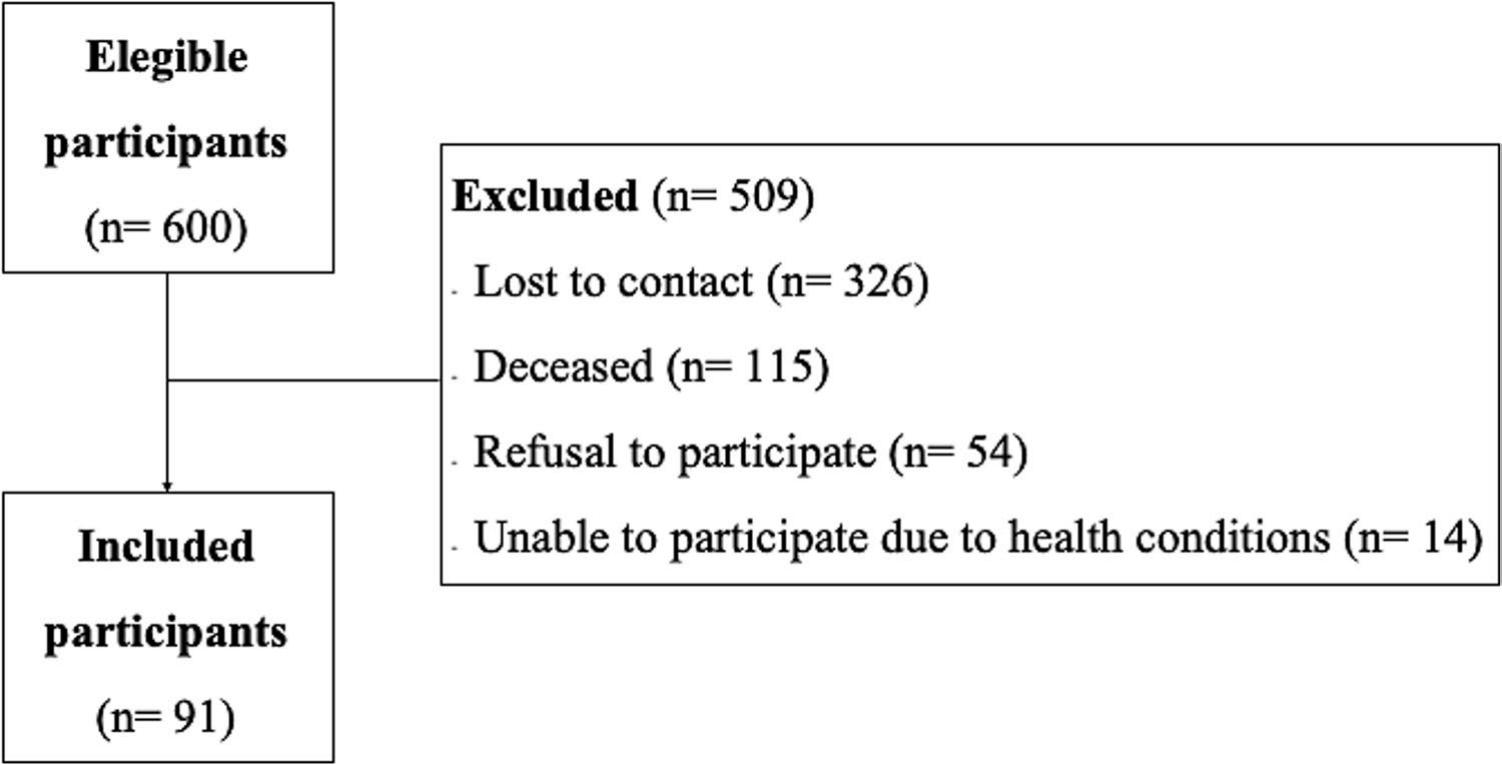
Study flow diagram

Figure 1 presents the participant flow diagram, including the number of eligible participants, the reasons for exclusion, and the final study sample.

### Sociodemographic characteristics

A total of 91 cancer survivors participated in the study: endometrial (*n* = 32), prostate (*n* = 18), breast (*n* = 20), and colorectal (*n* = 21). The mean age was 69 ± 9.24 years. Details are provided in Table 1.

**Table 1:** Sociodemographic characteristics of cancer survivors (breast, endometrial, prostate, and colorectal cancer)

Variables	*n*	%
	91	100
Sex		
Women	62	68.1
Men	29	31.9
Age group (years)		
40–49	4	4.4
50–59	10	11.0
60–69	36	39.5
70–79	31	34.1
80–89	10	11.0
Skin color		
White	33	36.3
Non-white	55	60.4
NR^a^	3	3.3
Marital status		
Without partner	46	50.5
With partner	45	49.5
Years of education		
0–9	39	42.8
10–12	32	35.2
> 12	20	22.0
Health insurance		
No	67	73.6
Yes	24	26.4
Income source		
Retirement/Other source of income^b^	56	61.5
Retirement + Paid work	14	15.4
Paid work	11	12.1
Paid work + Other source of income	3	3.3
No income	7	7.7
Income range (in minimum wages)^c^		
Up to 1	23	27,1
1–2	31	36,5
3–4	13	15,3
4–5	7	8,2
More than 5	11	12,9

^a^Not resported; ^b^Other source of income: Alimony, Bolsa Família, and other government social programs; ^c^Minimum wage (BRL 1518.00), *n* = 85, Don’t know/No response, *n* = 6

### Adherence to cancer prevention and surveillance recommendations

Table 2 describes the measures of adherence to cancer prevention and surveillance recommendations.

**Table 2:** Adherence to cancer prevention and surveillance recommendations

Variables	*n*	%
Dietary factor		
Daily intake of vegetables	39	42.9
Daily intake of fruits^a^	37	41.1
Daily intake of legumes	55	60.4
Weekly intake of red meet (> 500 g/day)^b^	63	70.0
Weekly intake of processed meet	27	29.7
Weekly intake of canned juices and powdered drink mixes	13	14.3
Weekly intake of soft drinks	28	30.8
Weekly intake of sweets^c^	38	42.7
Weekly intake of other ultraprocessed foods^d^	9	9.9
Alcohol intake	29	31.9
Tabacco use		
Current smoker	7	7.7
Former smoker	27	29.7
Never smoke	57	62.6
Physical activity^e^		
≥ 150 min/week	31	34.1
< 150 min/week	10	11.0
Do not practice physical activity	50	54.9
Screening and follow-up^f^		
Mammography (*n* = 35)	24	68.6
Cervical citology (Pap smear) (*n* = 45)	32	71.1
Colonoscopy (*n* = 88)	49	55.7
Prostate-specific antigen (*n* = 29)	24	82.8

^a,b^*n* = 90, Don’t know/No response, *n *= 1; ^c^*n* = 89, Don’t know/No response, *n *= 2;^d^ Other ultra-processed foods: instant noodles, ready-to-use seasonings, packaged snacks, frozen or ready-to-heat meals; ^e^According to WHO, 2020; ^f^According to current screening and surveillance recommendations

#### Dietary factors

Less than half of the population consumed fruits and vegetables daily. Among those who consumed these foods five or more times per week, none consumed them at least three times per day.

Regarding the intake of dietary factors associated with increased cancer risk, 70.0% reported consuming red meat in amounts exceeding the recommendation of up to 500 g per week, and 29.7% of the population consumed processed meat at least once a week. Sweet foods were part of the dietary routine for approximately 43.0%.

#### Physical activity behavior

Among the 91 participants, more than half did not engage in regular exercise or sports in the past three months, while only 34.1% performed more than 150 min per week, meeting the WHO recommendation for physical activity.

The majority (72.5%) reported having access to a public space near their home for physical activity. Although 60.4% were aware of public physical activity promotion programs, only 14.5% participated in them. The main reported barriers were program schedules incompatible with work or domestic activities (21.3%), lack of interest (19.2%), and health problems or physical limitations (23.4%).

#### Smoking

Former smokers had a median duration of exposure of 23.5 years (range: 2–57 years). Among current smokers, the median tobacco load was 3.8 pack-years. No participant reported using electronic cigarettes.

#### Sun protection

Approximately 19.8% of participants reported using sunscreen, and 34.1% used some form of protective accessory (such as sunglasses, hat, parasol, or protective clothing) during sun exposure.

### Screening and surveillance

Among participants within the recommended age range for mammography (50–69 years), the most frequently reported reasons for not having regular exams were long waiting times (27.3%) and the exam never being requested by a physician (27.3%).

Regarding women indicated for cervical cytology (Pap smear), the main reasons cited by those who did not have the exam regularly were not considering it necessary (16.7%) and the absence of a physician to perform it (16.7%). Furthermore, 41.7% reported being advised not to undergo the exam due to having had a hysterectomy or being over 60 years old.

Regarding colonoscopy, 40.4% had never done so. Among those who did not undergo the exam as recommended, 87.2% reported not receiving a medical request for it.

Concerning the prostate-specific antigen (PSA) test, indicated for men over 50 years of age, the reasons reported for not undergoing the test within the recommended period were long waiting times (33.3%), inadequate healthcare service (33.3%), and not considering it necessary (33.3%).

Additionally, the majority of participants underwent the aforementioned examinations through the Brazil’s Unified Health System (SUS).

### Health conditions and comorbidities

Table 3 presents information on the health conditions of the study population.

**Table 3:** Body mass index and health conditions of study population (*n* = 91)

Variables	*n*	%
BMI^a^		
Underweight	4	5.1
Normal weight	36	46.2
Overweight/Obesity	38	48.7
Comorbidities^b^		
Hypertension	62	68.9
Diabetes	29	32.2
Hypercolesterolemia	38	42.2
Cardiovascular disease^c^	6	6.6
Stroke	6	6.6
Arthritis	22	24.4
Chronic spinal disorders	31	34.4
Chronic lung disease	2	2.2
Chronic kidney disease	3	3.3

*BMI*, Body Mass Index. ^a^self-reported variable, *n* =78, Don’t know/No response, *n *= 13; ^b^n:90, Don’t know/No response, *n *= 1; ^c^ myocardial infarction, angina, heart failure, and arrhythmia

Among the comorbidities analyzed, hypertension was the most prevalent. 75.8% of participants visited a physician at least once a year for hypertension follow-up. Among those who did not attend healthcare services regularly, 66.7% did not consider this follow-up necessary. All hypertensive participants reported receiving a prescription for antihypertensive medication. However, 4.8% stated that they did not take all prescribed medications, and 1.6% reported not taking any. The reasons given for non-adherence to prescribed medications were lack of money to purchase them (25.0%), not considering it necessary (25.0%), and believing that their blood pressure was controlled (50.0%).

Among participants diagnosed with diabetes, the majority (89.6%) visited a physician regularly. The reasons reported by those who did not attend healthcare services regularly were long waiting times (66.6%) and absence of professionals or inadequate functioning of health units (33.3%). All diabetic participants used oral medication for glycemic control, and 14.3% also used insulin.

### Long-term sequelae resulting from cancer treatment

The long-term sequelae associated with cancer are presented in Table 4, along with their impact on participants’ daily activities.

**Table 4:** Reported late effects of cancer treatment

	*n*	%
Cognitive impairment^a^	39	42.9
Little impact in usual activities	4	10.3
Moderate impact in usual activities	9	23.1
Severe impact in usual activities	4	10.3
Hormonal symptoms^b^	36	39.6
Little impact in usual activities	4	11.1
Moderate impact in usual activities	4	11.1
Severe impact in usual activities	5	13.9
Sleeping disorders^c^	34	37.4
Little impact in usual activities	3	8.8
Moderate impact in usual activities	5	14.7
Severe impact in usual activities	6	17.6
Chronic pain^d^	27	29.7
Little impact in usual activities	2	7.4
Moderate impact in usual activities	6	22.2
Severe impact in usual activities	12	44.4
Urinary disorders^e^	24	26.4
Little impact in usual activities	5	20.8
Moderate impact in usual activities	4	16.7
Severe impact in usual activities	5	20.8
Bone disorders	23	25.3
Depression^f^	22	24.2
Little impact in usual activities	3	13.6
Moderate impact in usual activities	1	4.5
Severe impact in usual activities	5	22.7
Stress	21	23.1
Respiratory disorders^g^	19	20.9
Little impact in usual activities	6	31.6
Moderate impact in usual activities	3	15.8
Severe impact in usual activities	0	0
Lack of interest in activities^h^	15	16.5
Little impact in usual activities	1	6.7
Moderate impact in usual activities	4	26.7
Severe impact in usual activities	4	26.7
Fatigue^i^	14	15.4
Little impact in usual activities	2	14.3
Moderate impact in usual activities	3	21.4
Severe impact in usual activities	5	35.7
Sexual disorders^j^	16^ k^	64.0
Little impact in usual activities	0	0
Moderate impact in usual activities	1	6.2
Severe impact in usual activities	2	12.5
Lymphedema^l^	14	15.4
Little impact in usual activities	2	14.3
Moderate impact in usual activities	0	0
Severe impact in usual activities	2	14.3
Bowel disorders^m^	13	14.3
Little impact in usual activities	0	0
Moderate impact in usual activities	2	15.4
Severe impact in usual activities	3	23.1
Other health issues^n^	3	3.3

^a^No impact, *n* = 21; Don’t know/No response, *n* = 1; ^b^No impact, *n* = 22; Don’t know/No response, *n* = 1; ^c^No impact, *n* = 20; ^d^No impact, *n* = 7; ^e^No impact, *n* = 9; Don’t know/No response, (*n* = 1; ^f^No impact, *n* = 13; ^g^No impact, *n* = 10; ^h^No impact, *n* = 6; ^i^No impact, *n* = 4; ^j^Participants with active sexual life, *n* = 25; No impact, *n* = 11; ^k^Participants reporting sexual difficulties but without active sexual life, *n* = 2; ^l^No impact, *n* = 9; Don’t know/No response, *n* = 1; ^m^No impact, *n* = 7; Don’t know/No response, *n* = 1; ^n^Kidney pain, *n* = 1; hepatic steatosis, *n* = 1; hearing issues, *n* = 1)

Cognitive function alterations were considered concerning by 51.3% of those who reported them. Hormone-related symptoms, such as hot flashes, vaginal dryness, hair loss, and urinary incontinence, were considered concerning by 47.2% of those affected. Sleep-related disorders were considered concerning by the majority of participants who reported them (58.8%).

Chronic pain had a substantial impact on daily life and was considered concerning by 77.8% of those who reported it. Regarding treatment for symptom control, only 3.3% of participants used opioids. Additionally, 18.5% received specialized follow-up from healthcare professionals (e.g., physiotherapy, rehabilitation clinics, pain clinics), and 14.8% reported using non-pharmacological treatments (such as acupuncture, behavioral interventions, psychotherapy, relaxation techniques, or auriculotherapy). Other sequelae associated with cancer treatment are listed in Table 4.

Considering all long-term sequelae associated with cancer evaluated in this study, a total of 289 symptom reports were identified during the interviews (taking into account that a single participant could report more than one sequela). Despite this high number, seeking professional help to manage these effects was reported in only 77 cases (26.6%). Of these, 62.3% corresponded to treatment through a SUS healthcare professional, while the remaining 37.6% involved seeking care outside SUS. Difficulties in accessing any type of treatment was observed in 44.2% of cases.

Although no statistically significant association was observed between the types of treatment received and the reported sequelae, 38.7% (*p* = 0.01) of participants who underwent lymphadenectomy reported lymphedema. Information on participants’ cancer diagnosis and treatment are presented in Table 5.

**Table 5:** Tumour characteristics and treatment-related aspects of study population (*n* = 91)

Variables	*n*	%
Stage		
I	43	47.3
II	20	21.9
III	10	11.0
IV	1	1.1
Not specified	17	18.7
Surgery	86	94.5
Limphadenectomy	31	34.1
Radiotherapy	32	35.2
Brachytherapy	12	13.2
Chemotherapy	21	23.1
Hormoniotherapy	24	26.4

### Discharge from the CACON

Less than half of the study participants (38.5%) reported being referred to a PHC unit after cancer treatment. Receipt of a discharge summary to be presented to primary care providers, including guidelines on how health follow-up should proceed from that point onward, was reported by only 11 participants (12.1%). Of these, the majority (72.7%) indicated that the document was discussed at the time of delivery and that the information it contained was understood by them and their caregivers.

Additionally, 12 participants (13.2%) reported not attending the follow-up consultation prior to institutional discharge, becoming aware that their registration had been blocked at the institution, and consequently the end of oncology follow-up, through other means rather than through the consultation with the specialist physician.

Information related to cancer treatment was present in nine of the eleven discharge summaries analyzed. Only one of these included guidance regarding the frequency of routine consultations at the PHC unit, as well as laboratory tests, imaging exams, and signs or symptoms suggestive of cancer recurrence. Approximately seven summaries did not contain this information, and three patients reported being unsure whether such guidance was included in the document. Despite the observed incompleteness of the summaries, six participants who received them (54.5%) considered that the tool helped PHC professionals better understand the patient’s health status and, consequently, appropriately guide treatment based on specific needs.

Table 6 presents data related to access to health services after discharge from the CACON.

**Table 6:** Access to health services

Variables	*n*	%
FHS registration		
Yes	70	76.9
No	21	23.1
PHC team visit in the past year^a^		
None	39	55.8
Once	9	12.9
2–4 times	8	11.4
Bimonthly	5	7.1
Monthly	5	7.1
Don’t know/No response	4	5.7
Difficulty in scheduling medical appointments at the PHC^b^		
Yes	34	53.1
No	28	43.8
Don’t know/No response	2	3.1
Time to obtain a medical appointment at the PHC^b^		
Up to 30 days	18	28.1
31–60 days	17	26.6
61–90 days	9	14.1
≥ 90 days	15	23.4
Don’t know/No response	5	7.8
Feeling confident in the PHC professional’s care^b^		
Yes	41	64.1
No	18	28.1
Don’t know/No response	5	7.8

*FHS*, Family Health Strategy; *PHC*, Primary Health Care; ^a^*n* = 70; ^b^*n* = 64

## Discussion

Long-term cancer survivors require continuous follow-up, including clinical surveillance, psychosocial support, and actions aimed at health promotion and disease prevention. However, most previous studies in this population were conducted in countries without universal health coverage and with limited representation of obesity-related tumors. In this context, the present study aimed to describe access to primary health care, adherence to preventive and surveillance recommendations, and the prevalence of late effects among long-term cancer survivors. By considering the context of a public and universal healthcare system, this study contributes to the advancement of policies aimed at improving longitudinal and comprehensive cancer survivorship care. Furthermore, its findings may support the development of structured survivorship care plans, strengthening the counter-referral process to primary health care and enabling more coordinated and effective follow-up.

Participants in this study were predominantly older adults, without a partner, and with low educational attainment. Single individuals (including divorced and widowed) have a greater need for psychological support, which may negatively affect the care received [11].

In high-income countries, cancer survivors tend to have higher levels of education than those observed in the population of the present study [12–14]. Conversely, a recent study identified that patients with lower educational attainment show a higher prevalence of impaired functioning and severe symptoms [15]. Lower levels of education are associated with reduced access to health information, difficulties in understanding treatment, and lower adherence to recommendations for ongoing care.

### Adherence to cancer prevention and surveillance recommendations

Lifestyle-related cancer prevention strategies for survivors mirror those recommended for the general population, as they also help reduce the risk of developing other noncommunicable chronic diseases. The World Cancer Research Fund/American Institute for Cancer Research [16] recommends consuming at least five portions of fruits and vegetables daily, limiting red meat intake to no more than three portions per week, and avoiding ultra-processed foods, sugar-sweetened beverages, and alcohol. In addition, maintaining a healthy body weight and engaging in regular physical activity are emphasized. Adherence to these recommendations has been associated with reduced recurrence, lower risk of a second primary tumor, and decreased cancer-specific and overall mortality [17, 18].

Regarding dietary habits, less than half of the study population reported daily consumption of fruits and vegetables. Even among those who consumed these foods at least five times per week, none reported intake three or more times per day. Data from the Surveillance System for Risk and Protective Factors for Chronic Diseases by Telephone Survey (Vigitel), a nationwide telephone-based survey conducted periodically in Brazil, showed that the prevalence of recommended fruit and vegetable consumption in the Brazilian adult population ranged from 20.0% in 2008 to 22.1% in 2021, corroborating the low intake observed in the present study [19].

The consumption of ultra-processed foods and processed meats was similar to that reported in a study by Verde et al. (2025), which analyzed data from the Brazilian National Health Survey (PNS) on women with self-reported diagnoses of breast, ovarian, and cervical cancers. In that study, approximately 25.0% of participants reported consuming processed meats, soft drinks, industrialized fruit juice, cookies, and other sweets, while 6.4% consumed instant noodles and ready-to-eat foods. Moreover, red meat consumption was very frequent in our study, with more than half of the population exceeding the recommended intake.

Approximately 30.0% of the study population reported regular alcohol consumption. A study based on data from the National Health and Nutrition Examination Survey (NHANES 1999–2016) showed that cancer survivors consumed less alcohol compared with individuals who had never received such a diagnosis [20]. However, it is important to emphasize that there is no safe level of alcohol consumption for cancer prevention.

Regarding physical activity, only a small proportion of participants reported engaging in regular exercise, and fewer than half met the recommended 150 min per week. A similar finding was reported in a study with endometrial cancer survivors, in which only 30.0% of the sample was considered physically active [13]. Recently, a randomized clinical trial conducted with patients diagnosed with colon adenocarcinoma who had completed adjuvant chemotherapy showed that exercise significantly reduced the risk of recurrence, a new primary cancer, or death by 28.0% [18].

Regarding nutritional status, studies with cancer survivors show a predominance of overweight and a high prevalence of comorbidities, especially hypertension and diabetes, with a greater burden among patients with gynecological, colorectal, and urological cancers [13, 21]. In the present study, nearly half of the participants were overweight, 68.9% reported hypertension and 32.2% diabetes.

Comorbidities associated with metabolic syndrome highlight the importance of adequate management in the late cancer survivorship phase. Within the scope of the SUS, PHC has a crucial role in the follow-up and control of comorbidities. The organization and structuring of PHC teams are essential to face this challenge in a coordinated way, aiming for better outcomes [22]. Given the strong predominance of chronic diseases in the Brazilian population, fragmented health systems focused solely on acute care are unable to provide efficient, comprehensive, and high-quality care [23].

### Late treatment-related sequelae

The management of potential long-term sequelae should be a priority in the follow-up of cancer survivors, since adverse effects may arise or persist years after diagnosis and treatment [24]. Cognitive alterations, the most frequently reported sequela in the present study, include deficits in memory, reasoning, decision-making, processing speed, and concentration — characteristics of the so-called cancer-related cognitive impairment [25]. The severity of this impairment is significantly associated with a broader burden of clinical, demographic, cognitive, and psychological challenges [26], underscoring the importance of continuous surveillance and individualized care strategies for this population.

Regarding hormonal symptoms, the second most frequently reported sequela, a systematic review indicated that about 9.6% of women presented alterations in vaginal lubrication, with this condition being related to treatments such as surgery, radiotherapy, chemotherapy, and immunotherapy [27, 28]. Another symptom commonly associated with hormonal changes is alopecia, widely described in the literature as one of the most distressing side effects of cancer treatment. In women with breast cancer, for example, alopecia was reported as the third most undesirable adverse effect of chemotherapy, behind only nausea and vomiting [29].

Urinary incontinence, often linked to hormonal dysfunctions and the treatment of gynecological and prostate cancers, was also observed in our population. A study with endometrial cancer survivors who underwent brachytherapy and radiotherapy reported that the incidence of urinary incontinence affected more than half of participants [30], reinforcing the need for specific attention to this type of late effect in survivorship care.

Sleep disturbances in cancer survivors may exacerbate other late effects, such as fatigue, physical exhaustion, and cognitive impairment, underscoring the importance of their investigation [31]. Studies have reported that between 20.0% and 50.0% of survivors experience significant sleep disorders [32, 33], a finding consistent with the present study.

Our findings demonstrate how chronic pain can compromise quality of life, in line with a study that reported poorer quality of life among survivors with poorly controlled pain [34]. Similarly, another study identified that 9.5% of survivors reported pain related to cancer or its treatment, more frequently among those with multiple comorbidities [35].

Other late effects were also reported, including sexual dysfunction, depression, stress, breathing difficulties, fatigue, lymphedema, and bowel problems. Thus, understanding and monitoring these consequences are essential for the development of care strategies that aim not only to improve survival but also to enhance quality of life in the post-treatment period.

### Hospital discharge and transition of care to PHC

After institutional discharge, only a small proportion of participants received guidance on continuing follow-up through PHC. Failures in referral and counter-referral processes within the SUS can lead to the mistaken perception that this flow is limited to simple referrals between services [36]. A study on the breast and cervical cancer care pathway in Brazil identified precarious or even absent counter-referral [37], revealing a lack of coordination between primary care and specialized services, resulting in fragmented care.

Most survivors registered in the Family Health Strategy did not receive a visit from the Family Health team in the previous year. This gap in follow-up within the primary care network after institutional discharge can considerably affect the management of comorbidities and late effects of cancer treatment. In addition, the lack of structure, often reflected in prolonged waiting periods and insufficient staffing, can reinforce the perception of primary care as a place for occasional care rather than a true reference point after discharge, generating a reversal of roles within the health care network [38]. These factors, combined with structural deficiencies such as lack of equipment and supplies, have already been reported in the literature as compromising the care provided by PHC [39], potentially reducing trust in primary care quality and even leading to refusal of counter-referral within the health system. As a strategy to cope with the low resolution of health needs by PHC, individuals often turn to higher-complexity services, incorporating them as their primary point of care [38], which contributes to the overburdening of these units.

Failures in communication, especially during the transition of care, may contribute to increasing the sense of insecurity and vulnerability experienced by cancer survivors [40]. Therefore, the implementation of strategies that promote communication among services and interdisciplinary collaboration, as well as the sharing of responsibilities in care [38], are desirable. Among these strategies, survivorship care plans stand out, which should include a comprehensive summary of treatment and a follow-up plan for the individual [9], provided by oncology care providers.

The literature has evaluated the impact of using care plans in the follow-up of breast [41], prostate [42], endometrial [43], and colorectal [44] cancer survivors. However, to date, no studies have been found on the development and utility of such tools for the Brazilian population, considering the perspective of a public and universal health system. The development of this tool could be highly valuable for the care of survivors after cancer treatment.

Based on the data presented, this study provides evidence on transition to primary care, adherence to preventive and surveillance recommendations, and late effects among breast, endometrial, prostate, and colorectal cancer survivors in Brazil. However, some limitations should be acknowledged: the cross-sectional design, the limited sample size, recruitment from a single high-complexity cancer center, and the heterogeneity of tumor types and treatments received may limit the generalizability of the findings. Also, the use of telephone interviews and self-reported information may have introduced recall bias.

Furthermore, these findings may support the development of individualized survivorship care plans, including follow-up recommendations, guidance on potential long-term and late effects, and a definition of shared responsibilities between oncology and primary care teams. Such strategies may facilitate communication and counter-referral between levels of care, strengthen continuity of care after discharge from specialized services, and promote the timely identification and management of survivors’ long-term needs. However, further research is needed to better understand communication barriers between health services and to explore the perspectives of all stakeholders involved in survivorship care, thereby supporting comprehensive approaches that address the specific challenges experienced by this population.

## Supplementary Information

Below is the link to the electronic supplementary material.

ESM 1 (PDF 163 KB)

## Data Availability

The data used for this manuscript is available upon request to the corresponding author.
