# Supplementary material for: Transition from oncology to primary care: experience of long-term cancer survivors in a Brazilian high-complexity center
Source: Support Care Cancer. 2026 Jul 25;34(8):802. doi: 10.1007/s00520-026-11034-w (PMC13401524; doi:10.1007/s00520-026-11034-w)
Supplement: Supplementary file 1 — (PDF 163 KB) [file 520_2026_11034_MOESM1_ESM.pdf]

Article title: Transition from Oncology to Primary Care: Experience of Long-Term Cancer Survivors in a Brazilian High-Complexity Center

Journal name: Supportive Care in Cancer

Authors: Lidiane Araujo Cezário, Luiza Vianna Conteville, Juliane Alves da Silva, Gabriela Villaça Chaves.

Corresponding author: Gabriela Villaça Chaves
Divisão de Vigilância e Análise de Situação, Instituto Nacional de Câncer (INCA).
E-mail address: gchaves@inca.gov.br

Supplementary Table 1. Domain, dimensions, and references used for the development of the questionnaire.

Domains	Dimensions	References used as basis for questions at each dimension
Sociodemographic data	Sex and age	-
	Self-declared skin color	National Health Survey (*Pesquisa Nacional de Saúde*) (IBGE, 2019)[a]
	Education	National Health Survey (*Pesquisa Nacional de Saúde*) (IBGE, 2019)[a] / Demographic Census (*Censo Demográfico*) (IBGE, 2020)[b]
	Household information	National Health Survey (*Pesquisa Nacional de Saúde*) (IBGE, 2019)[a]
	Income and paid activities	National Health Survey (*Pesquisa Nacional de Saúde*) (IBGE, 2019)[a]
	Health insurance coverage	National Health Survey (*Pesquisa Nacional de Saúde*) (IBGE, 2019)[a]
Lifestyle and nutritional status	Anthropometric data and recent weight loss history	National Health Survey (*Pesquisa Nacional de Saúde*) (IBGE, 2019)[a] /Patient-generated Subjective Global Assessment (GONZALEZ et al., 2010)[c]

	Diet and alcohol consumption	National Health Survey (*Pesquisa Nacional de Saúde*) (IBGE, 2019)[a] /NCCN - Clinical Practice Guidelines in Oncology - Survivorship version 1.2021[d]
	Physical activity	National Health Survey (*Pesquisa Nacional de Saúde*) (IBGE, 2019)[a]
	Sun protection	NCCN - Clinical Practice Guidelines in Oncology - Survivorship version 1.2021[d]
	Smoking	National Health Survey (*Pesquisa Nacional de Saúde*) (IBGE, 2019)[a]
Health promotion, prevention, and surveillance in long-term survivorship	Health promotion, prevention, and access to health services	HODGKINSON et al., 2007[e] / CAMPBELL et al., 2014[f]
	Chronic health conditions	National Health Survey (*Pesquisa Nacional de Saúde*) (IBGE, 2019[a]; 2013[g])
	Late effects related to cancer treatment (pain, fatigue, lymphedema, cognitive function changes, sexual function changes, sleep disorders, hormonal, urinary and gastrointestinal symptoms, mental health changes)	NCCN - Clinical Practice Guidelines in Oncology - Survivorship version 1.2021[d]/HODGKINSON et al., 2007[e] / CAMPBELL et al., 2014[f] / SHAKEEL et al., 2020[h]

References: [a]INSTITUTO BRASILEIRO DE GEOGRAFIA E ESTATÍSTICA (IBGE). Pesquisa Nacional de Saúde 2019 (Questionário dos moradores do domicílio**)**. IBGE, 2019. Available at: https://www.pns.icict.fiocruz.br/wp-content/uploads/2021/02/Questionario PNS-2019.pdf. Accessed on: June, 2025; [b]INSTITUTO BRASILEIRO DE GEOGRAFIA E ESTATÍSTICA (IBGE). Censo Demográfico 2020 (Questionário Amostra). IBGE, 2020. Available at:https://www.ibge.gov.br/media/com_mediaibge/arquivos/bd918f26b77d18d86c251e7b1f7c1a70.pdf. Accessed on**:** June, 2025; [c]GONZALEZ, M. C et al. Validation of a Portuguese version of patient-generated subjective global assessment. Rev Bras Nutr Clin, v. 25, n.2, p. 102-108, 2010. Available at: http://www.braspen.com.br/home/wp-content/uploads/2016/12/02-Valida%C3%A7%C3%A3o-da-vers%C3%A3o-em-portugu%C3%AAs-da-avalia%C3%A7%C3%A3o-subjetiva-global-produzida-pelo-paciente.pdf. Accessed on: June 2025; [d]NATIONAL COMPREHENSIVE CANCER NETWORK. NCCN Clinical Practice Guidelines in Oncology: Survivorship. Version 1.2021. February, 2021; [e]HODGKINSON, K. et al. The development and evaluation of a measure to assess cancer survivors' unmet supportive care needs: the CaSUN (Cancer Survivors' Unmet Needs measure). Psycho-Oncology, v. 16, n. 9, p. 796-804, 2007, http:// doi.org/10.1002/pon.1137. [f]CAMPBELL, H. S. et al. Development and validation of the short-form survivor unmet needs survey (SF-SUNS). Supportive Care in Cancer, v. 22, n. 4, p. 1071-1079, 2014, http:// doi.org/10.1007/s00520-013-2061-7. [g]INSTITUTO BRASILEIRO DE GEOGRAFIA E ESTATÍSTICA (IBGE). Pesquisa Nacional de Saúde 2013 (Questionário dos moradores do domicílio). IBGE, 2013. Available

at: https://www.pns.icict.fiocruz.br/wp-content/uploads/2021/02/Questionario-PNS-2013.pdf. Accessed on: Dec 15, 2021. [h]SHAKEEL, S. et al. Evaluation of factors associated with unmet needs in adult cancer survivors in Canada. JAMA network open, v. 3, n. 3, p. e200506-, 2020, https//doi.org/10.1001/jamanetworkopen.2020.0506.
